# Advancing Strategies of Biofouling Control in Water-Treated Polymeric Membranes

**DOI:** 10.3390/polym14061167

**Published:** 2022-03-15

**Authors:** Hongli Zhang, Shilin Zhu, Jie Yang, Aijie Ma

**Affiliations:** 1 School of Materials Science and Chemical Engineering, Xi’an Technological University, Xi’an 710021, China; shilin971020@163.com; 2 School of Materials Science and Engineering, Xi’an Polytechnic University, Xi’an 710048, China; jieyang0320@126.com

**Keywords:** anti-biofouling, polymeric membrane, anti-adhesion, antimicrobial, hydrophilicity

## Abstract

Polymeric membranes, such as polyamide thin film composite membranes, have gained increasing popularity in wastewater treatment, seawater desalination, as well as the purification and concentration of chemicals for their high salt-rejection and water flux properties. Membrane biofouling originates from the attachment or deposition of organic macromolecules/microorganisms and leads to an increased operating pressure and shortened service life and has greatly limited the application of polymeric membranes. Over the past few years, numerous strategies and materials were developed with the aim to control membrane biofouling. In this review, the formation process, influence factors, and consequences of membrane biofouling are systematically summarized. Additionally, the specific strategies for mitigating membrane biofouling including anchoring of hydrophilic monomers, the incorporation of inorganic antimicrobial nanoparticles, coating/grafting of cationic bactericidal polymers, and the design of multifunctional material integrated multiple anti-biofouling mechanisms, are highlighted. Finally, perspectives on the challenges and opportunities in anti-biofouling polymeric membranes are shared, shedding light on the development of even better anti-biofouling materials in near future.

## 1. Introduction

Water is the source of human life. A sustainable high-quality water supply is essential for the rapid development of urbanization and industrialization [1,2]. However, due to the excessive use and increasing contamination of natural water sources, the scarcity of fresh water has evolved to be a global challenge [3,4]. In addition, the demand for drinking water quality has increased and the regulations on wastewater discharges have become a lot more rigorous. Therefore, to address the problem of water shortage while maintaining ecological and environmental well-being, considerable efforts are accordingly being made to develop various materials and technologies for the alleviation of the ongoing crisis by conserving the existing limited freshwater supplies and producing fresh water from abundantly available seawater.

Compared to the traditional water treatments techniques, such as distillation, extraction, electrochemical treatment, ion exchange, and adsorption, the emerging membrane separation process several merits: high removal efficiency, low economic and energy cost, smaller footprint size, chemical residual elimination, easy scalability, and environmental friendliness [5,6,7,8]. Reverse osmosis (RO) and nanofiltration (NF) are common membrane separation techniques for drinking water and wastewater treatments. Currently, thin film composite (TFC) structures, comprising a polyamide (PA) nanofilm layer on a supporting material, are state of the art technology for desalination by RO/NF. Currently, these advanced polymeric membranes, however, are inherently prone to fouling, especially biofouling, which dramatically deteriorates membrane permselectivity performance, thus increasing the operational cost and shortening the service life of the membrane. Biofouling is the major obstacle limiting the wide application of polyamide TFC RO/NF membrane in seawater desalination and wastewater treatment [9].

Fouling in RO/NF membrane can be classified into the following four types: inorganic/crystalline fouling, organic fouling (e.g., proteins, humic acid), colloidal/particulate fouling, and microbiological fouling (biofouling, adhesion, and accumulation of biofilm forming microorganisms) [10,11]. While the first three types of fouling can be eliminated by pretreated feed solutions or a periodic physical/chemical cleaning method, biological fouling cannot be fully removed by pretreatment or cleaning alone. Due to their self-reproductive nature, adhered organisms can grow, multiply, and propagate. Even if only a few microbial cells in water are survived after pretreatment, they will rapidly occupy the entire membrane surface, and multiply in the system [12,13,14,15]. Thus, the control of biological fouling becomes complicated and difficult. Therefore, addressing the intractable biofouling problem has become increasingly important.

To solve the problem, many strategies including using pretreatment processes, developing anti-biofouling RO/NF membranes, and combining cleaning methods have been proposed to alleviate or repair membrane biofouling. The objective of this article is to conduct a comprehensive and systematic review of surface modification tactics in polymeric separation membranes for inhibiting biological contamination on the basis of existing literature. Although anti-biofouling technologies and materials for RO/NF membranes have been extensively explored, there is still a lack of up-to-date and systematic reviews focusing on the control strategies and corresponding mechanisms of anti-biofouling. Therefore, there is a high demand for advancements to the recent developments in the control strategies and corresponding mechanisms of anti-biofouling in polymeric separation membranes.

**Figure 1:**
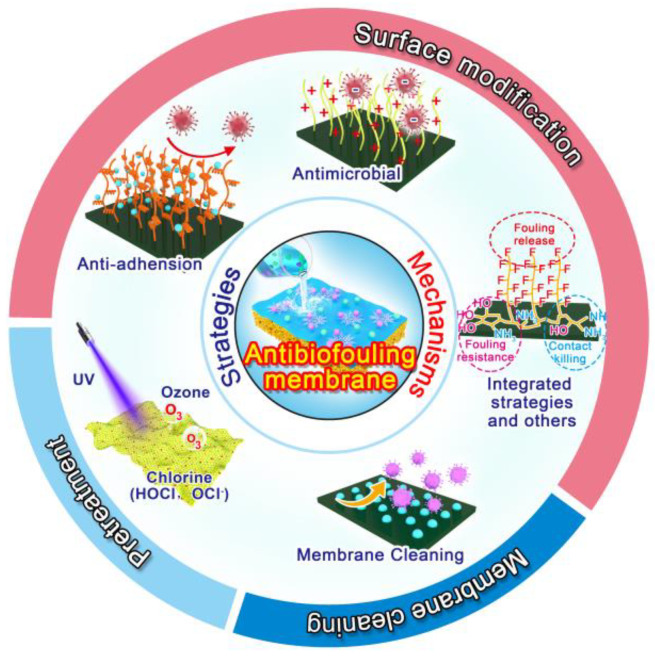
Schematic diagram of various strategies of biofouling control that are applied to water-treated polymeric membranes.

In this review, we briefly introduce the formation process, causes and influence factors of membrane biofouling. On this basis, current mitigation strategies of biofouling and the corresponding anti-biofouling mechanisms are systematically reviewed. Membrane microbial fouling mitigation strategies including anti-adhesion approaches, antimicrobial approaches, and versatile anti-biofouling materials that combine anti-adhesive and antimicrobial properties, were comprehensively summarized. Figure 1 represents the various strategies of biofouling control applied to water-treated polymeric membranes. Lastly, the challenges present in the usage of anti-biofouling tactics in practical water treatment applications and future perspectives were also discussed. This review can provide guidance for researchers and a valuable reference for the development of efficient anti-biofouling membranes/surfaces in the future.

## 2. Formation of Membrane Biofouling

Membrane biofouling is a complex and time-dependent phenomenon. It involves different phases to form a biofouling layer on a membrane surface. Separation membrane systems usually contain a large number of inorganic metal ions, organic macromolecules, particles, colloids, and microorganisms [16,17]. These pollutants are easily adsorbed or deposited on the membrane surface through strong physical and chemical interactions, which can destroy membrane structure and result in a significant decrease in membrane performance, thus increasing the operating cost and shortening the service life of the membrane, which restricts its wide application in the reclamation of wastewaters and treatment of industrial fluids [18,19].

**Figure 2:**
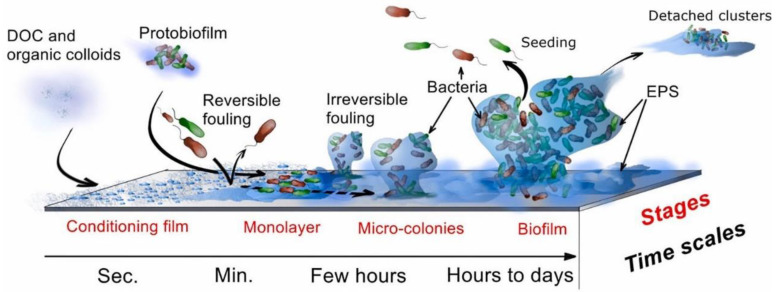
The sequence of stages leading to the formation of biofilm [33,34,35]. (Copyright (2015) with permission from American Chemical Society), (Copyright (2020) with permission from American Chemical Society), (Copyright (2014) with permission from Elsevier Ltd.).

Figure 2 shows the different stages of the formation of biological fouling on the membrane surface. It is proven that membrane biofouling originates from the initial physical adsorption or deposition of organic macromolecules/microorganisms on the membrane surface, followed by the colonization and proliferation of the adhered microbes, and eventually production of a bio-layer containing microbial cells and extra-cellular polymeric substances (EPS) on the membrane surface [20,21]. Even when the feed water contains only a minimal amount of microbial cells, once they have adsorbed onto the membrane surface, microorganisms will enter the system, adhere onto surfaces, start to grow, and multiply at the expense of the biodegradable organic matters dissolved into the ingested feed water or other dead microbial cells [22,23,24]. During reproduction and growth, the attached organisms excrete a gel-like structure of EPS secretions, which facilitates docking interactions for biofilm structures, and, together with microorganisms, forms a stable and difficult-to-degrade biofilm [25,26]. The biofilm matrix is a protective bacterial membrane that not only that can effectively resist the influence of external environment but participate dominantly in the membrane separation process. The rough viscoelastic surface of biofilm and EPS increase fluid frictional resistance and reduce the efficiency of convectional transport processes, causing the transmembrane pressure to drop and enhancing the concentration polarization effect. As a result, the permeate flux can be decreased dramatically and the salt rejection rate of RO/NF membrane can be deteriorated seriously [27]. Therefore, the effective inhibition of biological fouling has attracted tremendous interest in recent years and remains an alluring goal [28,29,30,31,32].

## 3. Influencing Factors of Membrane Biofouling

Membrane biological fouling starts with the attachment, sedimentation, and proliferation activities of microorganisms (*algae*, *protozoa*, *bacteria*, and *fungi*) and biofilm production on membrane surfaces [14,26]. The access and subsequent adsorption of microbes onto the membrane surface is a dynamic process and is relatively complicated [15,36,37,38]. Membrane biological fouling depends on a wide variety of physical and chemical factors that include microbial characteristics, membrane–microbe interaction, the properties of the membrane surface, operating conditions, and feed characteristics.

### 3.1. The Effect of Microbial Characteristics and Membrane–Microbe Interactions

It is previously proven that microorganisms can irreversibly adsorb onto the membrane surfaces through hydrophobic adsorption and electrostatic interactions. Firstly, the hydrophilicity/hydrophobicity of microorganisms affects the adsorption process, and hydrophilic surfaces tend to adsorb hydrophilic microbes. Additionally, the adsorption process of microorganisms is also influenced by the surface charges of the microbial cell, and the less the negative charge, the greater the corresponding adhesion force. The number of charged cells is strongly related to the pH values of the solutions, the ion concentrations, etc. Moreover, studies have shown that a high EPS content increases the probability that biological fouling will occur [39].

### 3.2. The Effect of Membrane Surface Properties

Membrane surface roughness, surface charge, and hydrophilicity greatly affect the adsorption of microorganism on membrane surfaces. At the initial stage, the organic macromolecules/microorganism’s adhesion to a membrane surface is significantly affected by the membrane surface roughness. A rough membrane surface forms a “valley,” which increases the adsorption surface area, thereby increasing the adsorption forces between the membrane and the microorganisms. The membrane surface charge characteristics also have the most important influence on the subsequent maturation of a biofilm [40,41,42]. The membrane surface is positively/negatively charged, causing the formation of a double electric layer on the membrane and microbial cells’ surfaces. This layer affects the adsorption process of microorganisms and facilitates the accumulation of cells on the membrane surface [43,44,45]. At the biofilm formation stage, the adhesion of the membrane surface is closely related to the membrane surface hydrophobicity, and the better hydrophilicity of the membrane surface, the stronger the antifouling performance is. Thus, the control of membrane biofouling could be achieved through the surface modification of commercial RO/NF membranes in order to reduce membrane surface roughness, increase the surface hydrophilicity, and to modify membrane surface charges that have the same electrical charge as the foulants.

### 3.3. The Effect of Operating Conditions and Feed Characteristics

Apart from the microbial and membrane surface’s properties, the operating environments and feed characteristics also accelerate membrane surface contamination to a certain extent [46,47,48,49,50]. Considerable efforts have been devoted to exploring the optimal conditions, but the optimal conditions are influenced by the treated water environments and membrane modules used in the market. As such, researchers have mostly focused on the components of feed water [11]. A high microbe content in water is likely to adsorb on the membrane surface, rendering the occurrence of biological fouling more likely. Furthermore, organic and inorganic substances in feed water also accelerate the process of adsorption of microorganisms and advance the biofouling layer formation [51].

## 4. Prevention and Control of Membrane Biofouling

To date, biofouling has been a major criticism of RO/NF membrane processes; the inhibition of the notorious biological pollution associated with these membranes is urgently needed. Several alternative strategies have been strongly proposed to inhibit the effect of biofouling [52,53,54,55,56]: (1) feed pretreatment carried out by using either biocide dosing or microfiltration(MF)/ultrafiltration(UF) membrane processes to physically remove organisms from the feed water of membrane systems, (2) membrane physical/chemical cleaning, and (3) surface modification of commercial TFC PA membrane.

Feed pretreatment by applying biocide dosing is the most direct pretreatment measure, and can reduce the content of the substances and bacteria which may cause membrane biofouling and facilitate the prevention of membrane surface biofouling. Unfortunately, these excessive flocculants and scale inhibitors also become the nutrient sources of microbial cells, and some bactericide-resistant microorganisms can survive in the water supply. In addition, the most commonly used bactericide, reactive chlorine, can lead to the destruction of membrane structures, especially for polyamide membranes, because of its strong oxidation. Membrane cleaning is an indispensable process for subsequent treatment, as it can alleviate the biological contamination of membrane surfaces to a certain extent, and therefore prolong the service life of membrane. However, unlike other sources of fouling, biofouling organisms have a self-replicating nature, since it is difficult to fully remove microbial cells by merely pretreatment (e.g., MF/UF or biocide application) or a periodic cleaning method [57,58].

Recently, a large number of surface modification approaches have been developed to enhance the anti-biofouling properties of membrane materials. As mentioned in an earlier section of this article, the important stages in biofilm formation are bacterial adhesion, microcolony formation, and biofilm maturation. Membrane surface modification is done primarily to prevent or slow one or more of these stages. Generally, the control of membrane biofouling can be mainly categorized into the following two approaches: anti-adhesion and anti-microbial. The anti-adhesion approach is to avoid the macromolecules’ attachment and microbes’ adsorption by constructing a surface with fouling resistance or fouling release properties. For instance, in one study zwitterionic and amphiphilic polymers were grafted or coated onto a membrane surface to achieve a hydrophilic and low surface energy structure [59,60]. The anti-microbial approach is to suppress the microbes’ growth and multiplication by using bactericidal agents [61,62]. The bactericidal strategy acts as a “safeguard” in order to kill the adhered microbes with bactericidal agents. Therefore, the fabrication of TFC PA membrane through the construction of anti-adhesive and anti-microbial surfaces, can fundamentally alleviate the membrane biofouling problem and become an effective way to inhibit the formation of biofilm fouling [63].

### 4.1. Anti-Adhesion Approaches

Bacterial adhesion has been found to decrease significantly by making the surface more hydrophilic, negatively charged, and/or smooth. Membranes with an anti-adhesive surface have been reported, including surface coating/grafting of hydrophilic monomers, zwitterionic/amphiphilic polymers, and incorporation of nanomaterials [64,65]. The polymer with hydrophilic groups facilitates the formation of a compact hydration layer through electrostatic interactions and hydrogen bonds, which can sufficiently prevent macromolecules and microbes from adsorption onto the membrane surface, reducing the irreversible membrane fouling [66,67]. The low surface energy structure can minimize the intermolecular forces between foulants and the membrane, thereby mitigating the extent of the reversible membrane fouling. Constructing anti-adhesive surfaces is still the most commonly applied solution to enhance the anti-biofouling effect of membranes.

#### 4.1.1. Hydrophilic Polymers

Hydrophilic polymers containing an abundance of polar groups are capable of forming hydrogen bonding interactions with water molecules in order to increase surface hydrophilicity and lower the number of interactions with nonspecific foulants [68,69]. Therefore, improving the hydrophilicity of the membrane surface can not only effectively reduce the adhesion of microorganisms and other contaminants, but also increase the permeating flux of the membrane. The highly hydrophilic polymers, such as polyethylene glycol (PEG)ylated materials, polyethylenimine (PEI), sericin (a natural polymer), hyperbranched poly(amido amine) (PAMAM), polydopamine, amino acids, and polyamide, have been commonly employed to combat nonspecific protein adsorption and cell adhesion due to their extremely low fouling ability [70,71,72,73].

D-amino acid (DAA) is an environmentally friendly biofilm inhibitor. Jiang et al. immobilized D-tyrosine on the polyethersulfone (PES) UF membrane in order to obtain a synergistic effect of surface hydrophilicity and anti-biofouling properties. The results showed that the grafting of D-tyrosine increased the surface hydrophilicity and endowed a smoother surface to the membrane. In addition, inoculation of the D-tyrosine PDA/PES membrane showed a better resistance to membrane fouling, paving the way for control of the biofilm growth and propagation [74]. Guo et al. synthesized a novel nanocomposite by combining DAA with polydopamine (PDA)-coated halloysite nanotubes (HNTs). The membrane modified with the nano-composite material was prepared by blending transformation, and the nano-composite material was evenly distributed in the modified membrane matrix [75]. Compared to the nascent membrane, the mechanical properties of the modified membrane are enhanced by the addition of nano-composite materials and excellent water flux and selectivity properties are achieved due to the improved hydrophilicity of the membrane. More importantly, according to static adsorption and dynamic filtration experiments with bovine serum protein (BSA), the resultant membrane obtained an outperforming anti-fouling ability. This study developed a novel and promising mitigation strategy for membrane biofouling.

Surface covalent grafting is a widely utilized method for surface hydrophilic modification because of its high performance stability and low risk of environment pollution. The modified substance reacts with the active sites on membrane surface, and therefore is anchored onto the membrane surface by covalent bonds, which has the merits of significant anti-adsorption and a long-lasting effect [76,77,78,79]. However, due to the harsh conditions of most chemical reactions and the use of strong polar solvents such as dimethyl sulphoxide, polymeric membrane surface structure tends to suffer damage, since it is highly desirable to explore a mild grafting reaction. The amidation reaction has been widely used in the graft modification process, owing to its mild conditions and high grafting efficiency. Kang et al. successfully grafted PEG and a series of derivatives onto RO membrane surface through an amination reaction. The hydrophilicity of the membrane surface was significantly improved and a better anti-protein adsorption capability was obtained [80]. Polyamide (PA) is a type of dendritic hydrophilic polymer, which has the high freedom to control the molecular polyamide amine chain proportion of hydrophilic chain segments with the aim of regulating the hydrophilicity of polyamide amine molecules. Dendritic polyamide can be successfully bonded onto the surface of the RO membrane through chemical reactions between the amine groups of polyamides and the residual chloride group of the nascent RO membrane. The incorporation of polyamide reduces the amount of negative charges on the RO membrane surface, and the resulting membrane exhibits a good fouling resistance to BSA adsorption [81].

Adjusting the surface charges and decreasing the roughness of the membrane is capable of improving the adhesion resistance and preventing microbial contamination. Xu et al. utilized polyethylenimine (PEI) with different molecular weight to regulate the surface charge of aromatic polyamide RO membrane, and the positively charged RO membrane was prepared through grafting PEI induced by carbodiimide [82]. The grafting of PEI effectively reversed the charges characteristics of membrane surface, and considerably improved the surface hydrophilicity of membrane, which not only compensates for the increase in hydraulics resistance caused by the attachment of the PEI layer, but also offers a superior fouling resistance against positively charged pollutants. Conclusively, the coating/grafting of a hydrophilic polymer is able to improve the membrane adhesion resistance in order to alleviate subsequent biological pollution to some extent.

#### 4.1.2. Zwitterionic/Amphiphilic Polymers

**Figure 3:**
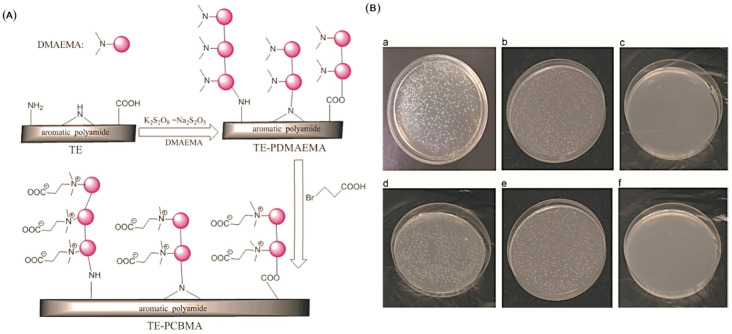
(**A**) The schematic of the membrane modification process. (**B**) a–c: The B. subtilis colonies after contacted with TE, TE–PDMAEMA10 and TE–PCBMA membrane, respectively. d–f: The *E. coli* colonies after contacted with TE, TE–PDMAEMA10 and TE–PCBMA membrane, respectively. (contact time: 2 h) [86]. (Copyright (2015) with permission from Elsevier Ltd.).

Zwitterionic polymers have also attracted extensive attention as a new generation of fouling resistant material in recent years [83]. The term “zwitterionic polymer” generally refers to the side chain containing both positive and negative charged units. There is a strong electrostatic force between zwitterionic polymers and water molecules. Compared with hydrophilic materials, zwitterionic polymers will form a stronger and more stable electrostatic force with water molecules, and thereby they have a better anti-adhesion effect for hydrophobic proteins and microorganisms. For instance, a zwitterionic polymer functionalized graphene oxide (GO) nanosheets was first synthesized and then incorporated into the thin layer of TFC PA membrane. Here, poly(2-(Methacryloyl)ethyl dimethyl-(3-sulfopropyl) ammonium hydroxide) (PMSA)-grafted GO (PMSA-g-GO) nanosheets served as an anti-fouling additive. The obtained thin film nanocomposite (TFN) RO membranes showed high hydrophilicity, improved permeation flux, and better resistance to BSA adhesion [84]. Yang et al. grafted a zwitterionic polymer, poly[(2-methacryloyloxy)ethyl]dimethyl[3-sulfopropyl] ammonium hydroxide (pMEDSAH), on the polyamide RO membrane surface via surface-initiated atom transfer radical polymerization (SI-ATRP). Specifically, RO membrane surfaces were firstly aminated with 3-aminopropyltrimethoxysilane (APTES), then the APTES layer was reacted with α-bromoisobutyryl bromide (BIBB), an acyl halide-type ATRP initiator. The grafting of the zwitterionic polymer, pMEDSAH, enhanced the surface hydrophilicity and decreased the membrane’s roughness, endowing a better resistance to bacterial adhesion. Meanwhile, in the process of cross-flow filtration, the modified membrane still maintains a good permselectivity performance after biological contamination [85]. Wang et al. modified the commercial aromatic PA RO membrane by the redox initiated graft polymerization of N,N′-dimethylaminoethyl methacrylater (DMAEMA), followed by the surface quaternization reaction with 3-bromopropionic acid (3-BPA) to obtain the zwitterionic carboxybetaine methacrylate (CBMA) polymer chains. The grafting of zwitterionic polymer changed the surface charge of the membranes. The modified TE–PCBMA membrane showed excellent resistance to the attachment of BSA and lysozyme(Figure 3) [86].

#### 4.1.3. Nanomaterials

**Figure 4:**
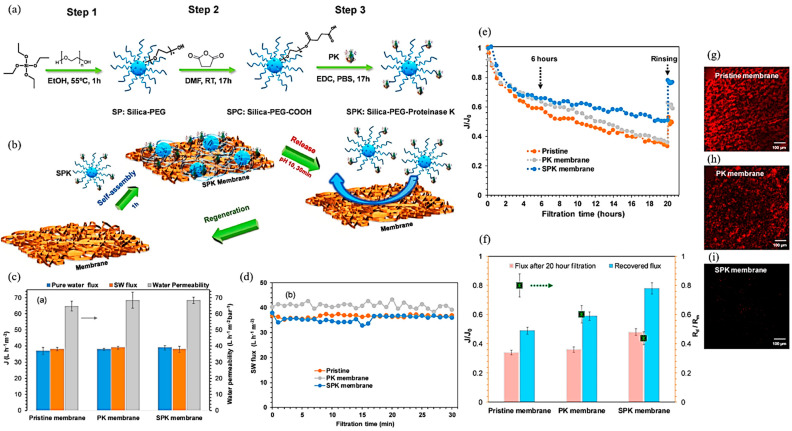
(**a**) Schematic diagram of the synthesis of SPK nanoparticles. (**b**) Schematic representation of the self-assembly and regeneration of SPK membrane. (**c**) Water permeability of the pristine, PK, and SPK membranes and their permeate fluxes for filtration of the pure water and municipal wastewater (absence of bacteria). (**d**) Fluxes over time of the pristine, PK, and SPK membranes during filtration of the municipal wastewater under Δ *p* = 0.5 bar. Pristine, PK, and SPK membranes’ filtration of *P. fluorescens* bacterial suspension (~10^7^ cell/mL) in municipal wastewater matrix. (**e**) Variation of filtration flux over time. (**f**) The operating fluxes after 20 h filtration, recovered fluxes after rinsing, and relative irreversible fouling resistance after filtration. (**g**–**i**) Fluorescence images of membranes after the filtration process, *P. fluorescens* (red) [87]. (Copyright (2021) with permission from Elsevier Ltd.).

The rapid growth in nanotechnology has spurred scientific interest into the environmental applications of nanomaterials. Particularily because of their exceptional structural characteristics, physicochemical properties, and environmentally benign nature, nanomaterials show great potential in water treatment processes. Inorganic nanomaterials were mainly used in UF/MF membrane in water treatment application, and research into RO/NF membranes was relatively late. Recently, because of their excellent hydrophilic and self-cleaning features, several nanoparticles such as TiO_2_, silicon dioxide (SiO_2_), magnesium oxide (MgO), copper oxide (CuO), and zinc oxide (ZnO) have been exploited for improving the adhesion performance of TFC PA membrane. Lan et al. proposed a simple strategy for the regeneration of a biology-resistant layer by self-assembling intelligent enzyme nanomaterials onto the commercial membrane surface to fabricate an anti-bacterial-fouling membrane [87]. These smart nanoparticles produced a fouling-resistant layer on the surface of a cellulose-based membrane by immersion method and the resulting membrane showed an excellent resistance to pseudomonas fluorescein biofilm. Filtration tests indicated that the SPK anti-fouling layer significantly improved the water permeation flux and effectively mitigated the membrane’s biological fouling (Figure 4). The doping of inorganic nanomaterials and surface functionalization, as used in the field of separation membranes, is an alluring issue and needs further exploration.

Based on the above discussion, an anti-adhesion effect can be achieved by constructing a surface with fouling-resistant properties. The coating/grating of hydrophilic polymers and inorganic nanomaterials is currently the primary choice to prevent the adhesion of proteins or microbes. Even though most of these hydrophilic surfaces can effectively resist the adsorption of proteins and bacteria, they do not deactivate the microbes, which is more important for combating biological fouling.

### 4.2. Antimicrobial Approaches

To inactivate the irreversibly adhered microorganisms and further inhibit the membrane biofouling, a bactericidal strategy is highly necessary. The anti-microbial strategy, by introducing antibacterial agents on the membrane surface, mainly serves to kill microorganisms adhering onto the membrane surface and to prevent the growth and multiplication of microorganisms to mitigate biological contamination of the membrane. Recently, considerable efforts have been devoted to developing novel antimicrobial agents to inhibit membrane biofouling, including silver/copper nanoparticles, emerging carbon-based nanomaterials, and cationic polymers such as quaternary ammonium compounds (QACs), guanidine derivatives, etc.

#### 4.2.1. Incorporation of Antimicrobial Nanoparticles (NPs)

**Figure 5:**
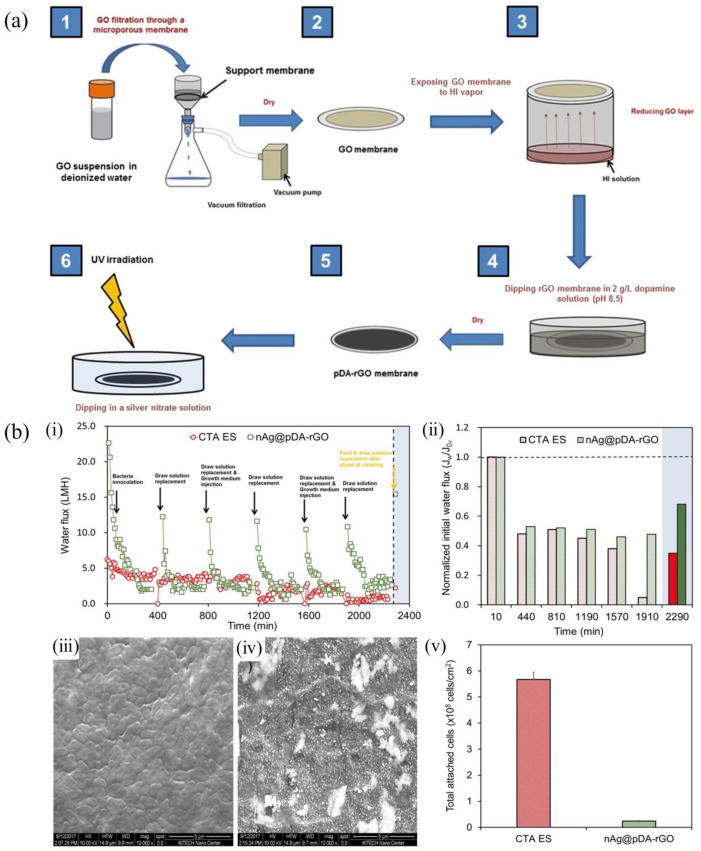
(**a**) Schematic diagram of the fabrication of nAg-pDA-rGO membranes. (**b**) ((**i**) Biofouling test in a cross flow FO filtration system showing water flux over time, (**ii**) normalized initial water flux, SEM images of commercial CTA ES (**iii**) and nAg-pDA-rGO membranes (**iv**) after the biofouling test, (**v**) the number of total cells attached on membranes [90]). (Copyright (2018) with permission from Elsevier Ltd.).

Incorporation of noble metal/metal oxide nanoparticles (NPs) onto membrane surfaces has been extensively utilized to prepare anti-biofouling TFC polymeric membranes [88]. As a biocide-releasing based material, silver NPs are one of the most studied bactericidal metal-based nano-antimicrobial material, exhibiting strong inhibitory and antibacterial activity towards a wide range of bacteria [89]. It is generally believed that the released metal ions (Ag^+^ or Cu^2+^) can interact with thiol (–SH) groups in microbial membrane cells and inactivate the proteins, which causes the leakage of phospholipids and phosphate in cells, destroys cell DNA replication, and controls the propagation of microorganisms. Yang et al. deposited silver nanoparticles (nAg) polydopamine (pDA) onto chemically reduced GO (rGO) laminates and the results showed high water flux and a strong resistance to biological contamination, providing insights into the development of new GO membranes for water purification (Figure 5a,b) [90]. Aymonier et al. mixed silver nanoparticles with a highly branching amphiphilic modified PEI, achieving a highly effective and environmentally friendly antibacterial surface coating. As a result, 98% fewer colonies were formed on the substrate coated with silver nanoparticles than on the substrate without the coating [91]. Zodrow et al. incorporated silver nanoparticles into a polysulfone UF membrane. The obtained membrane not only demonstrated antibacterial properties against a variety of bacteria, but also increased the membrane hydrophilicity, thus alleviating membrane biofouling [92]. Liu et al. prepared silver nanoparticles on an RO membrane surface through a reaction between dopamine and silver nitrate. The antibacterial rate of the obtained membrane against *Escherichia coli* reached 95.6%, and the antibacterial rate against *Staphylococcus aureus* reached 99.99% [93]. By slowly releasing bactericidal silver/copper ions which strongly bind to the bacterial membranes and inactivate proteins, the microorganisms adsorbed onto the membrane surface are eventually killed.

The membranes prepared with metal oxide nanometer particles, which are mainly represented by TiO_2_, ZnO, and CuO, show good bactericidal performance. TiO_2_ has been the focus of numerous investigations in recent years, particularly on its photocatalytic effects in the decomposition of organic chemicals and the killing of bacteria [94,95,96]. The release-based metal oxides (TiO_2_ NPs, etc.) are capable of generating various reactive oxygen species (ROS), such as the hydroxyl radical (OH), hydrogen peroxide (H_2_O_2_), etc., by reductive or oxidative reactions under light. These reactive oxygen species further destroy the outer membrane of the bacterial cells and eventually inhibit microbial proliferation. Pi et al. prepared PDA/PEI interlayer on polypropylene MF membrane using a co-deposition method, and then modified TiO_2_ nanoparticles on the membrane surface by a sol-gel process to prepare TiO_2_-modified membranes with different proportions. The results showed that the surface wettability and water permeation flux of the modified TiO_2_ NPs membranes were significantly improved, and that the modified TiO_2_ NPs membranes showed good anti-protein activity to BSA and lysozyme (Lys) [94].

Conclusively, metal/metal oxide NPs are currently used as the primary choice to fabricate antibacterial separation membranes. However, due to the face that it is attached onto the membrane surface merely by weak intermolecular interactions, the physical coating layer of biocidal nanoparticles is more easily exfoliated. According to the biocide-releasing mechanism, these highly toxic heavy metal NPs would uncontrollably leach from the membrane surface over time, gradually losing their antimicrobial activity and resulting in damage to the environment and human beings. Moreover, because of their widespread use, silver-resistant microorganisms have already emerged. Therefore, it is necessary to explore novel membrane materials with excellent antimicrobial properties and stability.

#### 4.2.2. Quaternary Ammonium Compounds (QACs)

Owing to their exceptional biocide efficiency, broad-spectrum antimicrobial activity, and long-term bactericidal stability, highly cationic polymers have attracted significant interest in recent years. The antibacterial activity of cationic polymers originates from the progressive attraction between the cationic polymers and negatively charged groups on the microbial cells’ membrane surface including phospholipids, proteins, and lipopolysaccharides. The strong electrostatic attraction disrupts or imposes a charge imbalance, which drives the breakdown of the cellular membrane and subsequently leads to the leakage of cellular content and eventual death of the bacteria.

**Figure 6:**
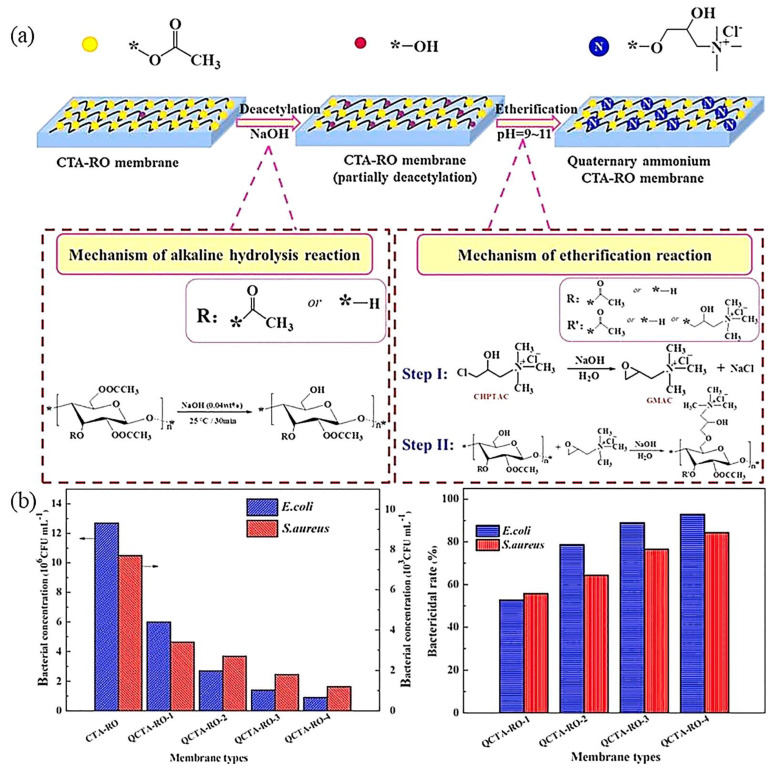
(**a**) Schematic diagram of the quaternization reaction between CTA-RO membrane and CHPTAC. (**b**) Measurement of the bacterial concentration and fungicide rate of the CHPTAC-modified membrane [99]. (Copyright (2018) with permission from Elsevier Ltd.).

Quaternary ammonium compounds (QACs) with long hydrophobic alkyl chains and cationic quaternary ammonium groups are more favored to construct antibacterial membrane surfaces because of their good resistance to protein adsorption and bacterial attachment [97,98]. Fei et al. introduced 3-chloro-2-hydroxypropyl trimethyl ammonium chloride (CHPTAC) onto a membrane surface and prepared a composite membrane with good bactericidal activity against Gram-negative *Escherichia coli* and Gram-positive *Staphylococcus aureus* on the membrane surface (Figure 6) [99]. The copolymers of sulfobetaine methacrylate (SBMA) and [2-(acryloxy) ethyl] trimethyl ammonium chloride (DAC) were grafted onto the surface of cellulose membranes, achieving satisfactory antifouling and antibacterial properties [100]. The relative molecular weight of the immobilized QACs on the membrane surface and the concentration of antibacterial groups were increased. As a result, the microbial cell could be inactivated in a short time. However, due to the complicated, time-consuming synthetic conditions and high-cost, it remains a challenge to create an antimicrobial PA membrane by surface covalently anchoring QACs [101].

#### 4.2.3. Guanidine Derivatives

Recently, polymers containing guanidine derivatives have demonstrated their great potential for combating bacterial pathogens and have attracted interest from the scientific community. In the molecular structure of guanidine functional groups, the positive charge is uniformly distributed around the central carbon atom and the three nitrogen atoms, resulting in an effectively resonant stable state of charged protons [102,103]. Moreover, the outer surface of a microbial cell membrane is negatively charged, while a mammalian cell membrane is electrically neutral. The positively charged substances kill bacteria through a strong electrostatic attraction with the surface of the bacterial cell membrane. Thus, guanidine compounds have a selective cell membrane, which reduces the toxicity of chemical reagents to mammalian cells. More importantly, the attraction between guanidine derivatives and the membrane surface sterilizes, so that the bacteria do not produce targeted specific binding and it is not easy for the bacteria to produce drug resistance [104,105]. The mechanism of the antibacterial activities by electrostatic attraction is very difficult to overcome for bacteria unless they change the structural properties of their cell membranes in long-term evolutionary processes. It is also worth noting that guanidines are highly soluble in water, enabling them having a very high acid dissociation constant in water, indicating that guanidine derivatives are better suited for stable electrostatic interaction with the anionic surface of microbes [106,107]. In summary, the guanidine-based polymers are superior to quaternary ammonium groups and have great application potential in antibacterial modification of membrane surfaces.

**Figure 7:**
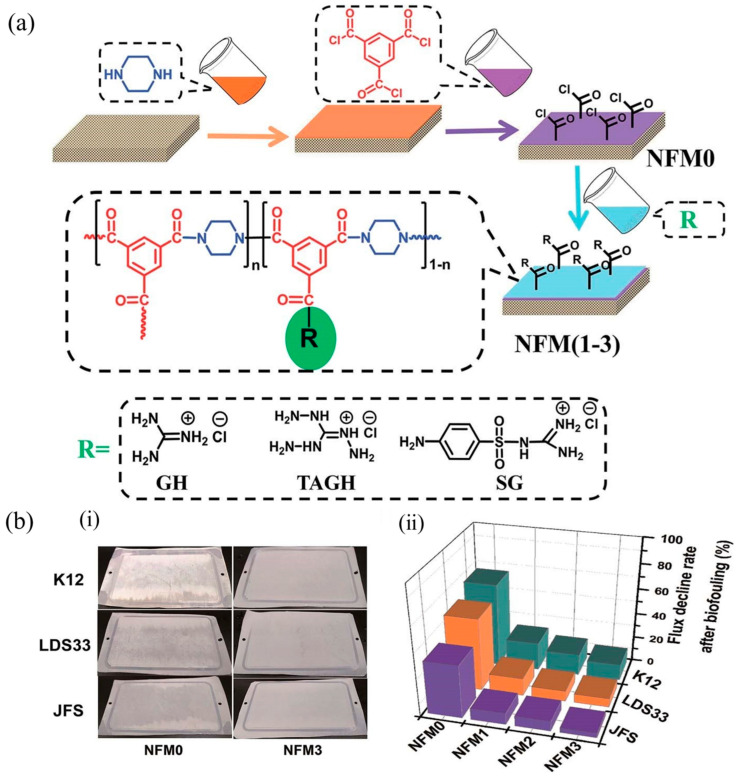
(**a**) Schematic diagram for the fabrication of guanidine-functionalized PA-TFC NF membranes. (**b**) ((**i**) Photographs of raw membrane (NFM0) and guanidine-functionalized membrane (NFM3) after 48 h exposure to K12, LDS33 and JFS suspensions and (**ii**) the water flux decline rate of NFMs after biofouling [109]). (Copyright (2018) with permission from Royal Society of Chemistry).

Li et al., introduced the synthetic guanidyl polythiamine (PVAMG) into a polysulfone carrier layer for polymerization reaction. The PVAMG modified RO membrane demonstrated an excellent bactericidal resistance against *Escherichia coli* [102]. Nikkola et al. coated the surface of a commercial RO membrane with a polyvinyl alcohol layer combined with polyhexa methyl guanidine hydrochloride (PHGH) to improve the membrane’s anti-biological fouling performance. The attachment of bactericidal PHGH not only improves the hydrophilicity of membrane surface and decreases the roughness, but also it endows good bactericidal activity against Gram-positive *Bacillus subtilis* and Gram-negative *Escherichia coli*. Unfortunately, the stability of the coating layer remains to be explored [63]. Zhao et al. modified RO membranes by grafting guanidine polymer (PEI-guanidine) onto polydopamine. The PEI-guanidine was formed by condensation of hydrochloride (GH), 1,6-hexanediamine, and polyethyleneimine (PEI), endowing the traditional RO membrane with excellent anti-adhesive and antibacterial properties [28,108]. Although PHGH is a promising bactericidal agent, its high synthetic cost, abundant consumption of regents, and insufficient quantity of hydrophilic groups strangle the application and construction of the anti-adhesion or antimicrobial surface. Previously, we constructed superior biofoulingresistant polyamide NF membranes via a convenient and effective strategy of separately pouring a series of small-molecule guanidine aqueous solutions including guanidine hydrochloride, triamineguanidine hydrochloride, and sulfaguanidine (SG) onto the surface of a traditional TFC polyamide NF membrane. The covalent attachment of guanidines significantly increased the hydrophilicity of the membrane surface, thereby the water flux was improved. The resulting guanidinium-functionalized NF membranes also simultaneously possessed fouling resistance and bactericidal attributes, endowing the membranes with excellent anti-adhesion and antimicrobial activities against Gram-negative *Escherichia coli* K12, Gram-positive *Bacillus pumilus* LDS33, and *Aspergillus parasiticus* JFS (Figure 7) [109]. On this basis, the co-solvent assisted second interfacial polymerization (CASIP) is introduced to fabricate a high-performance sulfaguanidine-modified PA NF membrane with a defect-free thin active layer, thereby favoring higher water permeance up to 79.0 L m^−2^ h^−1^ with a rejection rate above 98.3% for Na_2_SO_4_. CASIP includes the addition of a synergistic acetone solvent into the water phase. This membrane demonstrates enhanced anti-adhesive and antimicrobial performances against Gram-negative *Escherichia coli*, Gram-positive *Bacillus pumilus* LDS.33, and *Aspergillus parasiticus* JFS [110].

Additionally, the other polycations such as polyethylenimine derivatives and chitosan derivatives also exhibit potent antimicrobial activity via a membrane-lytic mechanism. The cationic compounds (quaternary ammonium salts) inhibit cell proliferation upon contact with microorganisms and have demonstrated a typical contact-killing antimicrobial attribute. The mechanism of anti-biofouling is absorption of the positively charged polymer molecules to the negatively charged microbial membrane via electrostatic interaction, hydrogen bonding force, and hydrophobic binding, which destroys the cell structure, causes the leakage of cytoplasm, and eventually leads to the cell’s death [111]. Although the contact-killing based materials exhibit potent bactericidal efficacy, the major concern is that the attachment of intracellular components or bacterial debris will eventually mask the active components and deteriorate the bactericidal performance.

### 4.3. Integrated Anti-Biofouling Strategies and Others

Membrane biological contamination is a multi-stage process; it includes the initial physical adsorption of an organic macromolecule, the adhesion of suspended bacteria, and the final colonization of the irreversibly adhered bacteria. In principle, any step that interferes with macromolecule adsorption, bacteria adhesion, and colonization can be regarded as a potential prevention strategy against biofouling. Considering the efficiency of a single/combined antifouling strategy and the complex foulants in the feed water, versatile NF membranes that integrate multiple anti-biofouling strategies with anti-adhesion and antimicrobial attributes is highly desirable [112].

**Figure 8:**
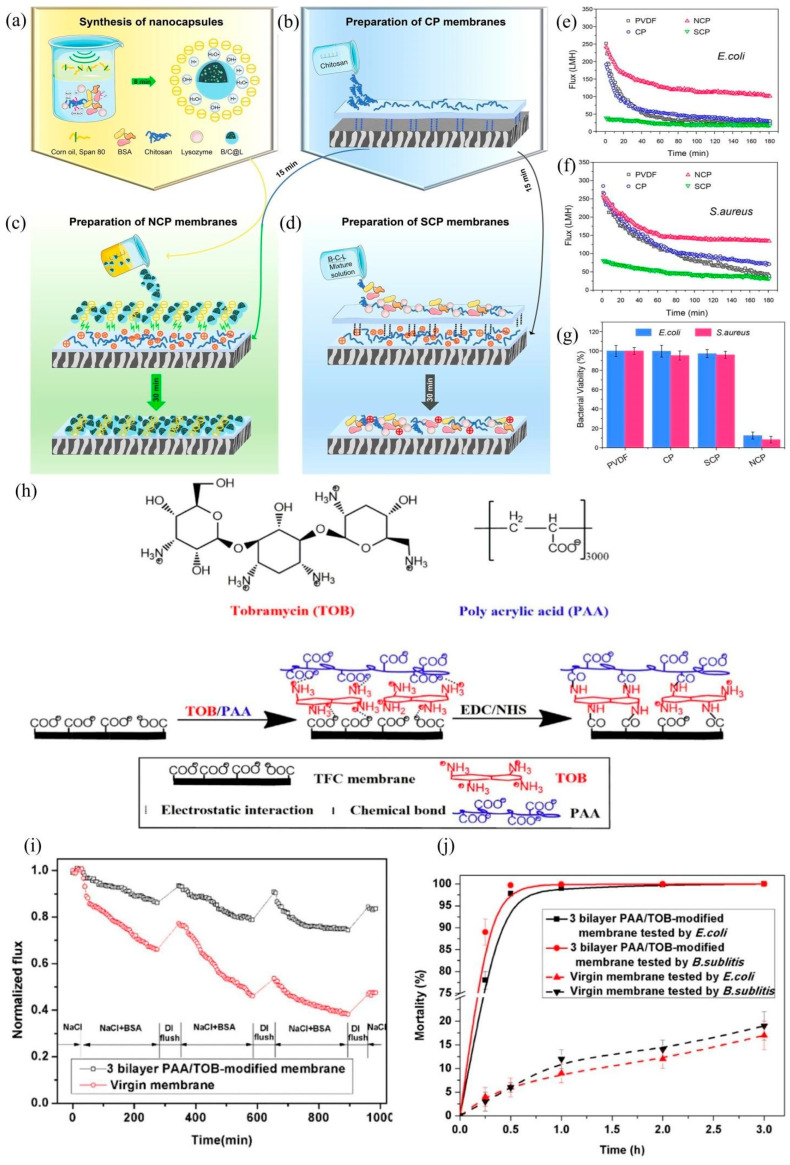
Schematic for the preparation of (**a**) B/C@L nanocapsules, (**b**) CP membrane, (**c**) NCP membrane, and (**d**) SCP membrane. The flux decline of pristine PVDF, CP, SCP, and NCP membranes during the (**e**) *E. coli* and (**f**) *S. aureus* biofouling experiment. (**g**) Bacterial viability of *E. coli* and *S. aureus* attachment to different membranes detected by the MTT assay. (**h**) Chemical structure of tobramycin (TOB) and poly acrylic acid (PAA), charged sites are labeled and the schematic diagram of the membrane modification process (**i**) Normalized flux of the virgin membrane and 3 bilayer PAA/TOB-modified membrane as a function of fouling time in the presence of BSA foulant (BSA: 100 ppm, NaCl: 2000 mg/L and pH: 7.0 ± 0.2). (**j**) The mortality of *E. coli* and *B. subtilis* for the virgin membrane and 3 bilayer PAA/TOB-modified membrane with contact time (incubation level: 10^9^ CFU/m^2^) [114,116]. (Copyright (2021) with permission from American Chemical Society), (Copyright (2017) with permission from Elsevier Ltd.).

Prince et al. immobilized the bactericidal silver NPs and then hydrophilic polyethylene glycol (PEG) on the surface of a polyethersulfone hollow fiber membrane. The prepared membrane, simultaneously possessed fouling release and fouling resistance features, shows an integrated anti-biological pollution ability [113]. Wang et al. introduced hydrophilic polyacrylic acid (PAA) and the bactericidal, tobramycin (TOB), onto the surface of a composite RO membrane by using a layered self-assembly process [114]. After modification, the roughness of the membrane was reduced, the hydrophilicity was significantly enhanced, and the permeation flux was increased by 18%. Thus, the membrane demonstrated good adhesion resistance, bactericidal characteristics, and stability (Figure 8h–j). Wang et al. successfully fabricated an RO membrane with triple antifouling attributes (fouling release, fouling resistance, and contact killing) via a surface chemical modification approach. The addition of 2,2,3,4,4,4-hexauorobutyl methacrylate (HFBM) with low-surface-energy brushes (fouling release), and hydrophilic TOB segments (fouling resistance) endows the membrane with superior antifouling properties. Further, owing to the bactericidal attribute of TOB, the membrane exhibits a strong antimicrobial activity [115]. These multifunctional anti-biofouling membrane materials have shown very good antibacterial activity, but they have inherent disadvantages such as their biocompatibility and biosafety, which have not been further studied.

Recently, antimicrobial enzymes (AMEs) and antimicrobial peptides (AMPs) have been regarded as potential alternative candidates to address the issue of membrane biofouling. In addition, the antibacterial activity of AMEs and AMPs has been attributed to the contact-killing mechanism. Unlike the cationic polymers, the biomolecules (AMEs and AMPs) normally have good biocompatibility. Li et al. developed a functionalized PVDF membrane with stimulus-responsive lysozyme nanocapsules (NCP). Coating and self-assembled nanocapsules were used to endow the membrane with improved lysozyme stability, anti-adhesion performance, and antibacterial activity [116]. As a result, the membrane showed excellent anti-biological contamination activity and the survival rate of bacteria was only 12.5%. The survival rate of bacteria on the filtered membrane was only 8.3%. The stimulated responsive lysozyme nanocapsule engineering MF membranes show great potential of anti-biological contamination in practical application (Figure 8a–g). Although the AMEs and AMPs achieve potent antibacterial activity and a low tendency to induce antimicrobial resistance, these materials have certain drawbacks including toxicity, short circulatory half-life (susceptible to proteolysis), and high manufacturing costs.

**Figure 9:**
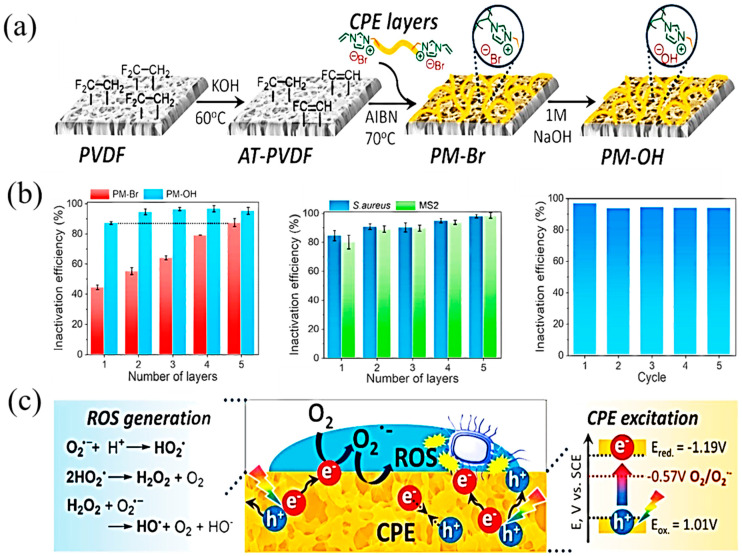
(**a**) Schematic illustration of synthetic procedure for the chemical tethering of CPE on a PVDF membrane to generate PMs. (**b**) Inactivation efficiency of *E. coli* on PMs with multiple CPE coating layers under visible light treatment for 1 h; Inactivation efficiency of S. aureus and MS2 on PM-OHs under visible light for 1 h; Cyclic experiment of *E. coli* inactivation over PM-OH-5L. (**c**) The proposed mechanism of antimicrobial activity on PM-OH [124]. (Copyright (2021) with permission from Elsevier Ltd.).

In addition, approaches involving the combined use of photoactive materials have gained significant attention and are promising. Early examples focused on the fabrication of photocatalytic membranes (PMs) with UV-responsive semiconductors, such as TiO_2_ and ZnO, and recently, visible-light responsive PMs were developed [117,118]. Conjugated polymers, consisting of π-conjugated polymeric backbone and ionic pendant groups, have a great potential to generate PMs with the advantage of visible-light absorbance, hydrophilicity, and functionality for covalent binding on membrane surfaces [119,120,121]. Jeong et al. demonstrated a visible-light-active photocatalytic membrane by grafting conjugated polyelectrolytes onto a commercial polyvinylidene fluoride (PVDF) membrane. The resulting hydrophilic PM exhibits excellent performance for photo degradation of organic dyes, photo-reduction of Cr(VI), and photocatalytic inactivation of mixed-culture biofilm under visible light irradiation. The anti-biofouling property enables > 97% flux recovery in repeated filtration cycles through the visible light treatment, even after it is fouled with a super-saturated bacterial feed solution (109 CFU/mL) (Figure 9) [122]. Ni et al. prepared novel PMs by modifying a hollow flower-like Bi_2_MoO_6_/CuS nanosphere. Compared with the nascent membrane, the photocatalytic-coupled separation membrane demonstrated increased hydrophilicity, improved permeability and a superior anti-biofouling performance under the irradiation of a light source [123].

Surface functionalization with carbon-based nanomaterials (CNMs) including 2D graphene and its derivatives, carbon nanotubes (CNTs), and carbon dots (CDs), shows promising antimicrobial activity on contact with bacteria [124,125]. Further, the synergistic effects have also been observed when combining CNMs with other antibacterial nanomaterials such as metal and semiconductor NPs, organic NPs, antibacterial polymers, and even antibiotics. These CNMs membranes mainly exert anti-biofouling effects by inducing membrane destruction mediated by physical disruption, charge transfer, the formation of reactive oxygen species, and the extraction of lipid from the cell membrane [13]. Even though dramatically increased antibacterial activities have been realized for CNMs through extensive research on their antibacterial effects, it is still too early to apply CNMs in practical commercial applications. These works provide valuable guidance for the coupling of membrane separation technology and photocatalytic technology. Conclusively, combining two or more anti-biofouling functionalities is considered to be most promising for antibacterial applications in the field of wastewater treatment.

## 5. Summary and Outlooks

Herein the recent advances in the development of anti-biofouling membranes for inhibiting membrane biological contamination was reviewed. This is of great significance to large-scale application of membrane separation technology in the field of water purification. Membrane biofouling in water environments is a complex and almost ubiquitous phenomenon. It involves different phases to progressively form a biofouling layer on the membrane surface. Biofouling is ascribed to the initial attachment or deposition of biological macromolecules/microorganisms, followed by the growth and multiplication of adhered microbes, and ultimately formation of a biofilm which seriously deteriorates the membrane permselectivity performance. Various reasonable and effective methods have been proposed to prevent and mitigate biological contamination in membrane systems. Neither feed pretreatment nor chemical cleaning technologies can fully remove microbial cells, which inevitably results in the destruction of polymeric membrane structures and create a regulatory risk in water treatment processes.

According to the hydrophobic adsorption and electrostatic interaction between microorganisms and the membrane surface, it is concluded that the anti-biofouling capability of a membrane directly depends on its membrane properties, such as surface hydrophilicity, roughness, and surface charge. Therefore, a large number of surface modification approaches have been developed to enhance the anti-biofouling properties of membrane materials. The two major approaches (anti-adhesion and anti-microbial) to combat surface biofouling are based on either preventing microorganisms from attaching or degrading them. Surface coating/grafting of hydrophilic monomers is the most commonly applied solution to inhibit the biofouling effect in membranes. Highly hydrophilic polymers, such as polyethylene glycol (PEG)ylated materials, polyethylenimine (PEI), sericin (a natural polymer), hyperbranched poly(amido amine) (PAMAM), polydopamine, amino acids, and polyamide, are capable of forming hydrogen bonding with water molecules in order to combat the nonspecific protein adsorption and cell adhesion. While most of these anti-adhesion surfaces may also resist the initial attachment of biofoulants, preventing the subsequent biofilm formation on membrane surfaces is difficult solely by anti-adsorption processes. In order to address the intractable problem of membrane biofouling it is highly desirable to design surfaces that are bactericidal.

Important strategies for killing or degrading bacteria include the design of surfaces that release silver or copper NPs, surfaces functionalized with polycations such as quaternary ammonium compounds (QACs), and guanidine derivatives. Noble metal/metal oxide NPs have long been used to construct surfaces that release biocidal agents. Because of their widespread use, silver-resistant pathogenic strains have already emerged. Moreover, silver-containing coatings act through a biocide-releasing mechanism and are therefore uncontrollably leached from the membrane surface over time. Surfaces functionalized with amphipathic polycations show promising antimicrobial activity; however, some modification processes require complex synthetic conditions, involve the use of dangerous and expensive chemicals, and are time consuming, thus increasing costs and may result in poor separation performance. Meanwhile, a single anti-microbial mechanism can only deal with a limited range of biofoulants. For complex contaminants (e.g., industrial wastewater, municipal sewage, and seawater), these designs have been insufficient. Thus, the synergetic effects of multiple anti-biofouling mechanisms should be considered as a superior strategy for the TFC polymeric membranes that are applied in the purifying and treating of wastewater containing more complex contaminants. Additionally, other techniques based on the use of carbon-based nanomaterials, enzymes, and photoactive agents are being investigated. Although the anti-fouling modification of polymeric separation membranes has achieved remarkable results, many problems remain to be further studied. These problems include new action mechanisms of microorganisms and membranes, key influencing factors on microbial growth and multiplication, and commercial applications. The development of surfaces that can inhibit the adsorption of biofoulants and novel types of anti-biofouling membranes is necessary.

Polymeric water-treated separation membranes dominate in the fields of desalination and wastewater treatment. The purpose of this review is to discuss the recent development of anti-biofouling membranes, which have been systematically reviewed in terms of their formation process, causes of membrane biofouling, membrane anti-biofouling modification strategies, and corresponding anti-biofouling mechanisms. Understanding the causes and mechanisms of microbial fouling is of great significance to the development of novel modified membranes for alleviating biofouling. In the future, more efforts should be made to develop novel types of anti-biofouling membranes. This review can provide some guidance for researchers and a valuable reference for the development of efficient anti-biofouling membranes/surfaces used in desalination, wastewater treatment, and purification processes.

## Data Availability

Not applicable.
